# Gut Microbiota is an Impact Factor based on the Brain-Gut Axis to Alzheimer’s Disease: A Systematic Review

**DOI:** 10.14336/AD.2022.1127

**Published:** 2023-06-01

**Authors:** Bin Zou, Jia Li, Rui-Xia Ma, Xiao-Yu Cheng, Rui-Yin Ma, Ting-Yuan Zhou, Zi-Qi Wu, Yao Yao, Juan Li

**Affiliations:** ^1^School of Pharmacy, Ningxia Medical University, Yinchuan 750004, China.; ^2^Department of Neurology and Clinical Research Center of Neurological Disease, The Second Affiliated Hospital of Soochow University, Suzhou 215004, China.; ^3^School of Basic Medical Sciences, Ningxia Medical University, Yinchuan 750004, China.; ^4^Ningxia Engineering and Technology Research Center for Modernization of Characteristic Chinese Medicine, and Key Laboratory of Ningxia Ethnomedicine Modernization, Ministry of Education, Ningxia Medical University, Yinchuan 750004, China.

**Keywords:** brain-gut axis, gut microbiota, Alzheimer’s disease

## Abstract

Alzheimer’s disease (AD) is a degenerative disease of the central nervous system. The pathogenesis of AD has been explained using cholinergic, β-amyloid toxicity, tau protein hyperphosphorylation, and oxidative stress theories. However, an effective treatment method has not been developed. In recent years, with the discovery of the brain-gut axis (BGA) and breakthroughs made in Parkinson’s disease, depression, autism, and other diseases, BGA has become a hotspot in AD research. Several studies have shown that gut microbiota can affect the brain and behavior of patients with AD, especially their cognitive function. Animal models, fecal microbiota transplantation, and probiotic intervention also provide evidence regarding the correlation between gut microbiota and AD. This article discusses the relationship and related mechanisms between gut microbiota and AD based on BGA to provide possible strategies for preventing or alleviating AD symptoms by regulating gut microbiota.

## 1. Introduction

Alzheimer’s disease (AD) is a degenerative disease of the central nervous system (CNS) closely related to age. It is mainly characterized by progressive cognitive decline and memory impairment, leading to defects in physiological function and making patients rely on caregivers [1]. With the aging of the population, it is important to explore AD pathogenesis and develop therapeutic methods. Although the onset process is slow, the disease continues to deteriorate from the initial short-term memory loss to behavioral problems such as language and orientation disorders and gradual loss of body function, eventually leading to death [2].

Observation results in the past 20 years show that gut microbiota regulates the development and function of immunity, metabolism, and nervous system through a dynamic two-way communication along the “gut-brain axis” [3]. These processes may contribute to the metabolic health of human hosts. They may lead to various common metabolic diseases in abnormal conditions [4], closely related to neuropsychiatric diseases, including AD. At the 34^th^ International Conference of Alzheimer’s Disease Association (AAIC) held on July 27^th^ to 31^st^, 2020, a special conference on the “Pathogenesis and Treatment of Microorganisms in AD” was held for the first time. The hypothesis of the interaction between microorganisms and CNS became one of the possible pathogeneses of AD. Therefore, understanding the function of gut microbiota and its relationship with AD is very important for studying AD pathogenesis.

## 2. Brain-gut axis

The brain-gut axis is the connection network involving multiple biological systems. It establishes the two-way communication between the gut microbiota and brain and maintains the dynamic balance of the intestinal tract, CNS, and microbial system [5, 6]. Communication pathways in these biological networks include direct and indirect signal transmissions through chemical transmitters, neural pathways, and immunoregulation [3]. The intestinal tract community (intestinal flora) represents the maximum density and abundance of microorganisms in the human body. The bacteria in the intestinal tract are equivalent to the number of human cells [7], and the number of genes they encode is about 150 times that of the human host [8], which significantly expands the metabolic potential of human beings [3,9]. The gut microbiota is essential for immune system function, metabolism, and even the development of various organs [3,10,11]. The intestinal bacterial community is located at the intersection of the host and environment. As a channel connecting the intestinal tract with other organs, including the brain, gut microbiota may affect human health in many aspects.

**Figure 1.**
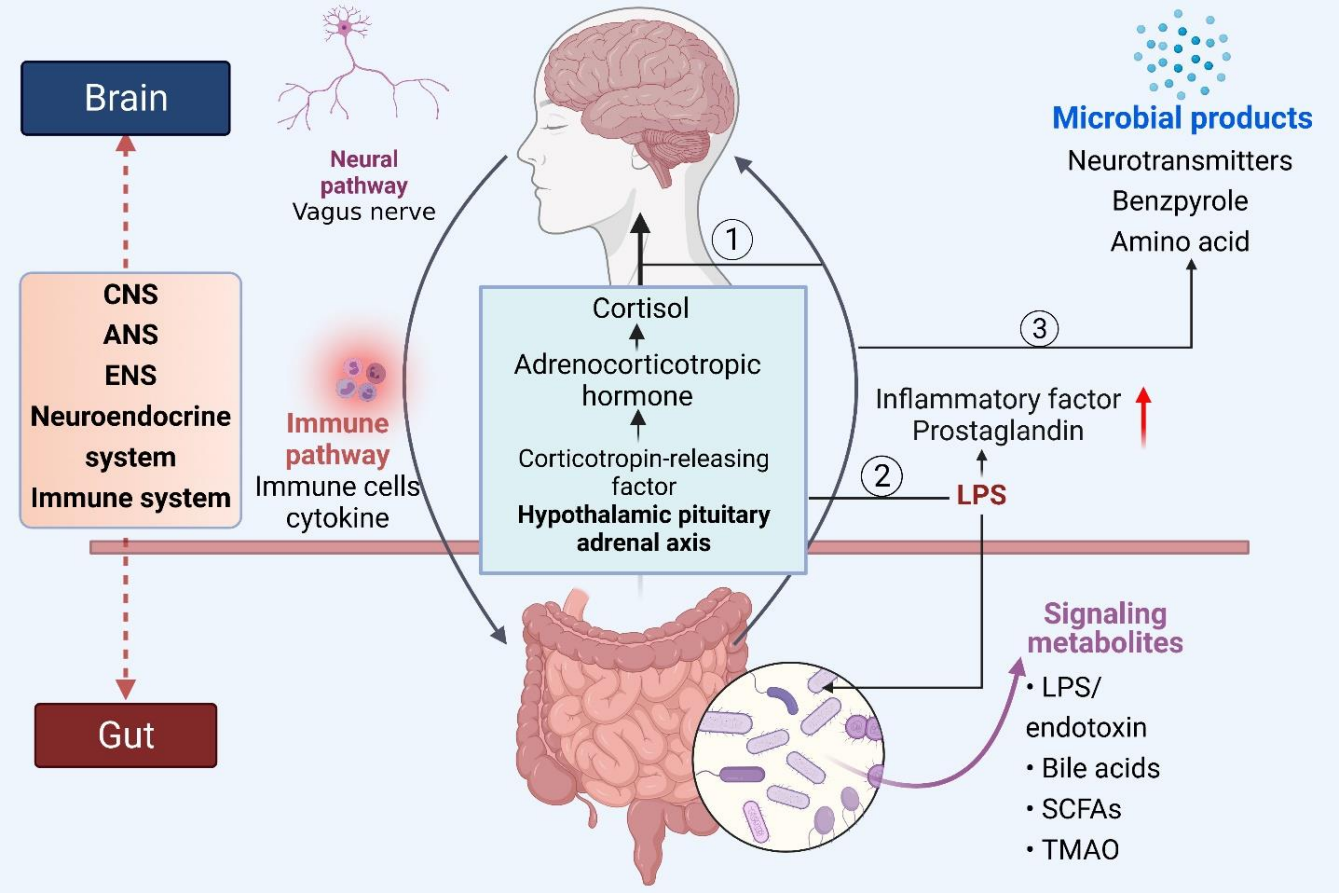
**Schematic representation of the mechanisms involved in the crosstalk between the gut microbiota and brain.** Abbreviations: CNS = central nervous system, ANS = autonomic nervous system, ENS = enteric nervous system, LPS = lipopolysaccharide, TMAO = trimethylamine N-oxide, and SCFAs = short-chain fatty acids.

In the 1880s, W. James and C. Lange first proposed that the two-way communication between the CNS and intestinal organ plays a role in emotional regulation [12,13]. Forty years later, Walter Cannon put forward that the brain plays an important role in regulating intestinal function [14]. Several studies have proved that the flora, intestine, and brain are closely related, with frequent interaction [8,15-17]. Therefore, this special communication pathway is called the “brain-intestine axis.” The gut-brain axis is a communication network for information exchange between the intestine and the brain, including CNS, autonomic nervous system (ANS) [18], enteric nervous system (ENS), neuroendocrine system, and immune system [19,20]. The interaction mechanism between gut microbiota and the brain is shown in Figure 1. Several direct and indirect ways of the intestinal-brain axis mediate two-way communication between intestinal microflora and CNS [3,16].

On the one hand, the CNS affects intestinal permeability and motility through the immune pathway (inflammatory cytokines) and nerve pathway (vague nerve) from the brain to the intestines. It changes the structure and composition of intestinal flora [21] and affects the functions of the nervous system, endocrine system, and immune system [22]. Intestinal flora can activate the hypothalamic-pituitary-adrenal axis (HPA) to cause cortisol release from the intestines to the brain, controlling the activation state of microglia and affecting the inflammatory state of the brain [23]. Secondly, intestinal microbial disorder promotes endotoxins (such as LPS), inflammatory cytokines, and prostaglandins production, directly affecting neuro-inflammation or activating peripheral immune cells [24]. Thirdly, intestinal flora produces or stimulates the release of many metabolites, such as indole, neurotransmitters, and amino acids, which enter the blood and brain to regulate neurons, astrocytes, microglia, and the blood-brain barrier [25,26]. Animal experiments showed that the gut microbiota could change the neuronal circuit through signal transmission mechanisms and modulate the structure and function of neurons through neurotrophic substances. These changes affect the development and behavioral function of the normal brain [9,27]. Ingestion of probiotics can change the balance of intestinal microorganisms, leading to changes in intestinal microbial content, which impact the host CNS, immune system, and endocrine system [28].

## 3. Gut microbiota

Microbes are closely related to animals and human beings. Microorganisms include bacteria, archaea, fungi, and viruses, collectively called microflora [3]. Studies on human microbial flora in the past ten years have shown that endogenous intestinal flora is essential for maintaining human health. The endogenous intestinal flora’s structure, quantity, distribution, and biological characteristics are constantly changing [1]. Intestinal flora comprises trillions of microorganisms, including at least 1000 different bacteria. It is considered the largest microbial repository in the human body, accounting for about 95% of bacteria in adults [29]. The Human Microbiology Project Alliance published the dominant bacteria of human intestinal flora at the phylum, class, and family levels in 2012 (Table 1).

**Table 1:** Dominant bacteria of human intestinal flora in different levels of classification.

Biological classification of bacteria	Dominant bacteria	References
**Phylum**	*Bacteroides, Firmicutes, Proteobacteria, Actinobacteria, Cyanobacteria*	[18, 19, 29, 146]
**Class**	*Clostridia, Gammaproteobacteria, Bacteroidia, Bacilli, Negativicutes, Actinobacteria*	[45]
**Family**	*Lachnospiraceae, Ruminococcacea, Enterobacteriaceae, Bacteroidaceae, Veillonellaceae, Erysipelotrichaceae, Enterococcaceae*	[45]

Under normal dynamic balance conditions, the healthy intestinal tract has a relatively stable symbiotic microflora [14]. The intestinal flora is non-pathogenic and has many beneficial effects on the physiological activities and nutrition of the human body. For example, intestinal flora participates in energy metabolism, reduces inflammatory response, and stimulates systemic immunity. Furthermore, it protects the host from pathogen invasion and infection, promotes the absorption of nutrients, and participates in the production of amino acids. It also promotes intestinal motility and protects intestinal mucosa, which is extremely important to the growth and health of the host [30].

The imbalance of intestinal flora may lead to different diseases, including AD. The gut microbiota imbalance is closely related to gut barrier dysfunction and intestinal permeability. Gut barrier dysfunction can release microbial metabolites into the circulatory system. In the case of blood-brain barrier (BBB) leakage, several pro-inflammatory cytokines in circulation can enter the central nervous system and induce neuro-inflammation via activating microglia and astrocytes [31,32]. In addition, the imbalance of intestinal flora reduces the intestinal barrier integrity, making it easier for pathogens to invade [33]. Cani et al. observed an increased intestinal permeability in rats treated with a high-fat diet. It may be due to the increased bacterial endotoxin level and increased inflammatory cytokines such as TNF-α and IL-1β. Oral antibiotics could effectively normalize intestinal flora diversity and intestinal permeability [31,34]. The imbalance of intestinal microbiota contributes to neuro-inflammation, which plays a key role in the pathogenesis of AD. In AD pathogenesis, the intestinal flora imbalance changes intestinal permeability and induces inflammation in the intestine. It also induces inflammation in the central nervous system, as pro-inflammatory cytokines can enter the bloodstream and affect the brain [14,31,35,36]. Lastly, the intestinal flora imbalance may aggravate Aβ accumulation. Minter et al. found that mixed antibiotic treatment could reduce Aβ plaque load in APP/PS1 mice [31,37].

## 4. Relationship between gut microbiota and AD

The treatment with *Clostridium butyricum* could improve the cognitive function of rats, decrease neuronal apoptosis, and increase concentrations of brain-derived neurotrophic factors. It also reduces the production of pro-inflammatory cytokines and exerts a neuroprotective effect [44]. Zhuang et al. found that specific bacterial populations, including *Bacteroides* and *Actinomycetes*, in the intestine of AD patients were significantly increased compared with that of the control group [45]. Clinical research shows that the abundance of two anti-inflammatory bacteria, *Bacteroides fragilis*, and *Eubacterium rectale*, is lower in the intestinal tract compared with pro-inflammatory bacteria (e.g., *Escherichia coli* and *Shigella*) in elderly patients with cognitive impairment [39,46]. The increasing number of pathogenic bacteria and the abundance of *E. coli/Shigella* are positively correlated with the increased expressions of pro-inflammatory cytokines (interleukin IL-1 and CXCL2) and NLRP3 inflammasome [47]. Some microorganisms in the intestinal tract release LPS, amyloid, and other exudate complexes and immunogenic mixtures from their adventitia to the surrounding environment. These changes promote Aβ accumulation and inflammation, activating the signal pathway in the pathogenesis of AD. Bacterial amyloid protein is related to molecular and cellular adaptation, adhesion stimulation, aggregation, biofilm formation, tissue invasion, bacterial colonization, and pathogen infectivity.

*E. coli*, *Salmonella*, *Mycobacterium*, *Bacillus subtili*s, *Mycobacterium tuberculosis*, and *Staphylococcus aureus* are some strains producing functional extracellular amyloid fibers [48]. *E. coli* endotoxin can promote the Aβ fiber formation *in vitro*, inducing the occurrence and development of AD [48]. Curli is the most studied bacterial functional amyloid. The effective adhesion of *E. coli* to host cells and tissues mediated by curli facilitates cell invasion and pathogenesis. It reveals curli as an important bacterial factor in human-bacterial interactions. Curli is recognized by the immune recognition of Toll-like receptors (TLRs), as shown by recent studies. This triggers a cascade that leads to caspase activation, pro-inflammatory chemokines and interleukins release, and autoantibodies production. This autoantibody production favors bacterial infection, causing perturbance of the host immune system and self-damage to the host [49].

Previous studies found that the gut microbiota diversity of AD patients was significantly decreased when 16S rRNA gene sequencing was used to classify and identify the composition of intestinal flora in fecal samples of individuals with and without AD [18,50]. Cattaneo et al. analyzed the fecal microbiota of 40 amyloid-positive patients with cognitive impairment, 33 amyloid-negative patients with cognitive impairment, and the control group individuals. The results showed that the numbers of *E. coli* and *Shigella* in the amyloid-positive group with cognitive impairment increased compared to the other two groups. In contrast, *Acinetobacter rectum* and *Bacteroides fragilis* decreased [47]. The changes in intestinal flora in AD mice and patients at different classification levels are shown in Table 2.

**Table 2:** Changes of intestinal microflora in AD mice and patients.

Biological classification of bacteria	Increased	Decreased	References
**Phylum**	*Bacteroidetes, Proteobacteria*	*Firmicutes, Verrucomicrobia, Actinobacteria*	[54, 136, 147, 148]
**Family**	*Enterococcaceae, Lactobacillaceae, Veillonellaceae, Rikenellaceae, Gemellaceae*	*Lachnospiraceae, Bacteroidaceae, Bifidobacteriaceae, Ruminococcaceae, Clostridiaceae, Mogibacteriaceae, Turicibacteraceae, Peptostreptococcaceae*	[45, 54, 147, 149]
**Genes**	*Doreia, Bifidobacterium, Streptococcus, Acinetobacter, Ruminococcus, Coprococcus, Blautia Escherichia, Shigella*	*Bacillus, Succinivibrio, Sutterella, Prevotella, Pasteurella, Clostridium, Bifidobacterium, Lachnospira, Oxalicobacteria, Alistipes, Bacteroides, Alloprevotella, Haemophilus, Paraprevotella, Barnesiella, Butyricimona, Akkermansia, Allobaculum, Blautia, Ruminococcus*	[41, 54, 136, 147, 148, 150]

Vogt et al. observed that YKL-40, a biomarker in cerebrospinal fluid (CSF) of AD patients, was positively correlated with the numbers of *Bacteroides* and *Clostridiaceae* [50]. YKL-40 is a secreted glycoprotein mainly expressed by astrocytes in the brain. It is elevated in various neurological conditions and is a general marker of neuroinflammation. It activates the innate immune system and cell processes related to extracellular matrix remodeling [51,52]. The YKL-40 expression increases in reactive astrocytes and microglial cells during neuro-inflammatory processes. Several studies have found that YKL-40 is increased in the CSF of patients with mild cognitive impairment and AD and cognitively normal individuals with amyloid pathology.

Moreover, YKL-40 levels are elevated in autosomal dominant AD mutation carriers 15 to 19 years before estimated symptom onset, shortly after the beginning of brain amyloid accumulation [51]. Shen et al. compared the fecal microbiota composition of elderly and middle-aged groups in southwest China. The results showed that the contents of *Coprococcus* and *Sutterella* were significantly higher, while those of *Lachnospira* and *Oxalicobacteria* were lower in the elderly [53]. Scott et al. evaluated the relationship between behavior, physiology, and cecal microbiota in aged mice. Compared with younger mice (2-3 months old), aged mice (20-21 months old) showed more obvious spatial memory impairment, anxiety-like behavior, and higher intestinal permeability. The abundance of *Porphyromonas* and *Streptomycaceae* also significantly increased [53]. The above results suggest that intestinal microbiota is closely related to AD.

Liu et al. identified the fecal microbiota of patients with AD, patients with amnesia mild cognitive impairment (aMCI) before onset, and normal cognitive health control (HC). The characteristics of fecal microflora in patients with aMCI and AD were reported and compared with those of healthy elderly people in China for the first time. The results showed that the fecal microflora diversity of AD patients was lower than that of aMCI and HC individuals. The fecal microbiota composition in AD patients changed, characterized by a decrease in the number of colonies. It produced short-chain fatty acids (SCFAs) and rich *Proteus* that promoted inflammation, which was related to AD severity. Compared with pre-dementia aMCI and healthy individuals, some microbial communities, especially *Enterobacteriaceae*, could be associated with AD patients. These findings may promote the understanding of AD pathogenesis and provide new strategies for diagnosing and treating the disease [54].

## 5. Mechanism of gut microbiota in AD

### 5.1. Metabolite

The metabolites of intestinal microbiota affect the host’s neurophysiology in various ways, such as blood circulation, humoral pathways, the immune system, and neurons [46,55]. Several bacterial components and metabolites that significantly affect AD pathogenesis are discussed below.

#### 5.1.1 LPS

Lipopolysaccharide (LPS) is a large glycolipid from the outer membrane of *Gram-negative*, which is formed by the partial connection of lipids and polysaccharides by covalent bonds. It plays the role of endotoxin and is a powerful trigger of innate immune system response, leading to inflammation [56]. Zhao et al. first reported the presence of LPS, a marker of chronic inflammatory disease, in the hippocampus and superior temporal lobe neocortex of patients with AD [57]. Compared with the age-matched control group, the average LPS level of the cerebral neocortex in elderly AD patients increased by 26 times. The plasma LPS concentration was three times higher than that in normal individuals [57]. As the patients age, the intestinal microbiota diversity and the pathogenic bacteria level decrease. These changes increase chronic inflammation, decrease anti-inflammatory bacteria levels in the intestine, and increase LPS production. LPS can activate microglia, changing the blood-brain barrier integrity, increasing intestinal permeability, causing a higher level of inflammation in the CNS, and accelerating the neurodegenerative disease process [58]. Animal experiments also confirmed that intraperitoneal injection of LPS could increase the β-amyloid protein level in the hippocampus of mice, resulting in learning disabilities [59].

#### 5.1.2 Trimethylamine N-oxide (TMAO)

**Figure 2.**
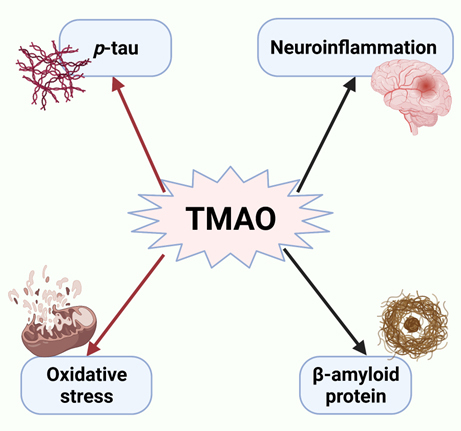
**Role of TMAO in the development of Alzheimer’s disease.** TMAO can increase central nervous system inflammation, β-amyloid protein, and tau protein accumulation. Tau protein in cerebrospinal fluid is closely related to TMAO, and it can increase oxidative stress and damage mitochondrial function, resulting in cognitive impairment. TMAO = trimethylamine N-oxide.

Trimethylamine N-oxide (TMAO) is a small molecule produced by intestinal microbial metabolism [60], which can promote neuro-inflammation and the accumulation of β-amyloid and tau proteins by inducing the imbalance of intestinal microorganisms. It also exerts a series of pro-inflammatory and pathogenic functions [61]. Vogt et al. detected the TMAO and AD biomarkers levels in CSF of AD patients, mild cognitive impairment (MCI) patients, and normal controls. The expression levels of *p*-tau and *p*-tau/Aβ42 in patients with AD and MCI were significantly higher than those in the control group, accompanied by neuronal degeneration. However, they were not correlated with Aβ42/Aβ40, indicating that the relationship between TMAO and tau pathology is closer than that of amyloid deposition alone [60]. In addition, Vogt et al. detected the biomarkers of neuronal degeneration in CSF. It was found that TMAO was related to the increase of neurofilament light chain protein but not to neurogranin, suggesting that TMAO is related to axonal injury but not to dendritic degeneration [60]. TMAO causes neurodegeneration by affecting fragile neurons rather than amyloid production. In the brain, TMAO induces neuronal aging, increases oxidative stress, damage’s mitochondrial function, inhibits rapamycin target protein (mTOR) signaling [62], and can lead to brain aging and cognitive impairment (Fig. 2). Therefore, pharmacological preparations that inhibit the production of TMAO can be developed to slow the course of AD [63].

#### 5.1.3 Amino acid

Amino acids in the form of protein are a large part of the human diet and can be used as potential energy sources. The most abundant amino acids in the human brain include glutamic acid and glutamine, followed by aspartic acid, taurine, serine, γ-aminobutyric acid, and glycine [64]. Disorders of amino acid and nitrogen metabolism are associated with neurological defects and, in some cases, dementia. Studies have shown changes in serum and brain amino acid levels in AD patients and AD model mice [65]. Glutamate is an excitatory neurotransmitter, and one of its metabolites, γ-aminobutyrate (GABA), is an inhibitory neurotransmitter. Therefore, the changes in glutamate metabolism in the AD brain significantly affect neural function [65]. In astrocytes, GABA can be synthesized from arginine. An increase in the GABA level synthesized through this pathway plays a role in cognitive impairment [66]. Tryptophan (Trp), the precursor of serotonin formation in the brain, is an essential amino acid derived from the diet. Most Trps are metabolized by the kynurenine pathway (KP) through the canine pathway or by intestinal microflora to indole. The metabolites of Trp can regulate intestinal immune cell function through aromatic hydrocarbon receptors [58,67]. Microflora significantly affects the host immune response by regulating Trp [39]. Therefore, the loss of Trp reduces 5-HT and leads to cognitive decline [68].

Similarly, intestinal bacteria affect the diet’s metabolism of tyrosine and valine. Tyrosine is an essential amino acid and the precursor of catechol neurotransmitters dopamine (DA), norepinephrine, and epinephrine [69]. These tyrosine-dependent neurotransmitters affect various central and peripheral functions, especially the DA neurons in the prefrontal cortex, which are involved in stress response and working memory. Stress state and aging are characterized by neurotransmitter depletion and behavioral and cognitive impairment. Since the tyrosine content *in vivo* is easily changed by dietary intake, cognitive function can be recovered by increasing tyrosine intake [70]. Valine is a branched-chain amino acid derived from dietary protein, and the brain is an important branched-chain amino acid reactor. Branched-chain aminotransferases (BCATs), which catalyze the decomposition of branched-chain amino acids, maintain the neurotransmitter supply and several expressions in the brain’s glial cells.

Recent advances in metabonomics have shown that the metabolic changes of branched-chain amino acids are associated with AD progression. Lower plasma valine levels are associated with accelerated cognitive decline, and valine concentrations are significantly lower in AD patients. Conversely, higher valine concentrations can reduce AD risk as the brain more easily absorbs valine than other branched-chain amino acids [71]. Therefore, several researchers have studied the relationship between tyrosine, valine, and intestinal flora. Some studies have found that lactic acid bacteria *Bifidobacterium* and *Bacteroides* affect the accumulation of tyrosine in feces [72], while *Lactobacillus* and *Corynebacterium* can produce valine [73]. Therefore, the changes in these bacteria affect the accumulation of tyrosine and valine and impact cognitive function [68,74,75].

By detecting the metabolites in the feces of 5×FAD mice with non-targeted metabonomics, Wang et al. found significant changes in amino acid pathways, especially phenylalanine-related pathways. Analysis of amino acids in mouse blood showed that phenylalanine, isoleucine, histidine, and acetylornithine had higher concentrations, especially phenylalanine and isoleucine. Furthermore, their transporter Slc7a5 was expressed in Th1 cells. The results showed that phenylalanine and isoleucine were easily absorbed by Th1 cells and significantly promoted the differentiation and proliferation of Th1 cells. In the case of gut microbiota imbalance, it will accumulate phenylalanine and isoleucine in peripheral blood and feces. It will stimulate the differentiation and proliferation of pro-inflammatory peripheral infiltrating cells and aggravate the neuro-inflammation and cognitive impairment, as the Th1 immune cells are related to the activation of M1 microglia. In the fecal transplantation (FMT) experiment, the feces of 2-month-old WT mice significantly decreased the concentrations of phenylalanine and isoleucine in 5×FAD mice. After intraperitoneal injection of phenylalanine and isoleucine in WT mice, the number of Th1 cells in peripheral blood increased. These findings emphasize the role of intestinal microorganisms’ abnormal production of phenylalanine and isoleucine in inducing neuro-inflammation dominated by Th1 cells. It suggested that they are strongly related to the progression of AD [38].

One study conducted a metabonomic analysis of the CSF of MCI patients, AD patients, and age-matched healthy controls. The amino acid levels in AD patients and mice’s CSF, blood, and feces were changed. The results showed that the metabolites dimethylarginine, arginine, valine, proline, serine, histidine, choline, creatine, carnitine, and glycine might be biomarkers of disease progression [65] (Table 3). However, the plasma amino acid levels show great variability, depending on the amount of fasting the subjects experienced before donating blood [65].

Increasing or decreasing dietary levels of specific amino acids and other metabolites improves markers of aging and longevity [65]. However, several obstacles must be overcome before effective treatments can be developed. These include studying intestinal transport, bioavailability, liver metabolism and excretion, and blood-brain barrier transport to select the best formula [65]. This information is presented in a few commonly studied amino acids but is missing in most amino acids [65]. Liver metabolism seems to be a huge challenge to overcome. Although large amounts of amino acids are added to treat neurodegeneration, intestinal transport in the elderly may also become limited [65,76].

**Table 3:** Altered amino acid levels in AD mice and patients.

Amino acid	Brain	Serum	Feces	References
**Valine**		Decreased	Increased	[65, 68, 74]
**Phenylalanine**	Increased	Decreased	Increased	[38, 65]
**Tryptophan**	Increased	Decreased		[65, 68]
**Tyrosine**		Decreased	Increased	[65, 72]
**Glutamine**	Increased	Decreased		[65]
**Glutamic acid**	Decreased	Increased		[72]
**Lysine**	Decreased			[72]
**Histidine**		Decreased		[72]
**Cysteine**	Increased	Decreased		[65, 72, 151]
**Methionine**	Increased	Decreased		[72]
**Isoleucine**		Increased	Increased	[38]
**Aspartic Acid**	Decreased			[65]

Dietary amino acids can provide the human body with a large amount of carbon and nitrogen, which can be metabolized through various biochemical pathways. Therefore, amino acids play an important role in neuronal signal transduction, energy generation, production, and removal of nitrogenous waste [65]. The brain and serum of patients with AD show many changes in amino acid levels and metabolism, which provide the basis for some disease symptoms and valuable insight into possible drug discovery targets in AD research [65].

#### 5.1.4 Bile acid

Bile acid (BA) is the product of cholesterol metabolism and clearance. It is synthesized in the liver as the “primary BA” [77]. After the primary BA is transported to the gallbladder and stored, it is secreted into the small intestine and metabolized by intestinal bacteria into secondary BA. The coupled primary BA is decomposed and uncoupled by bacterial bile salt hydrolase (BSH) [78]. In a rat brain, 20 kinds of BA have been identified, including nine unbound BA and 11 conjugated BA [79]. Most of these BAs exist in rat blood [80]. BA can also be synthesized in the brain or transported from the peripheral circulation to the brain through BA transporters [81].

Primary BA, secondary BA, and conjugated BA show different affinity with BA receptors. Binding BA is selective to the receptor and requires transporters to cross the BBB. On the contrary, lipophilic unbound BA may cross the BBB through passive diffusion [82]. BA can phosphorylate occludin (one of the BBB membrane proteins) through ras-related C3 botulinum toxin substrate 1 (RAC1), increasing the permeability of BBB directly [81,83,84]. Meanwhile, intestinal BA is found to control the growth and reproduction of bacteria to maintain the barrier’s permeability [81]. BA can affect the function of various neurotransmitter receptors, including M2, M3 acetylcholine receptors, GABA, and N-methyl-D-aspartate receptors. M2 receptors are distributed throughout the brain and are essential for cognitive function. M3 receptors are located in neurons that project to areas such as the hippocampus and substantia nigra [81,85]. Therefore, BA affects cognitive, memory, and motor functions [81,86].

A recent study reported the association between intestinal microbiota, BA distribution, and genetic variation in AD pathogenesis. Compared with controls, serum BA profiles in AD patients changed, hepatogenic primary BA significantly decreased, and secondary binding BA and their conjugates, glycine deoxycholic acid (GDCA), taurodeoxycholic acid (TDCA), and glycolithocholic acid (GLCA), produced by bacteria increased. The higher the level of secondary conjugated BA, the worse the cognitive function [61,87]. GDCA and TDCA, which bind to secondary BA, activated the M2 receptor and affected cognitive function [81]. Therefore, supplementation of primary BA can improve cognitive impairment.

Some studies have shown that conjugated BA Tauroursodeoxycholic acid (TUDCA) reduces the accumulation of Aβ peptide in the hippocampus and frontal cortex and ameliorates memory impairment of APP/PS1 double knockout mice [88,89]. Ursodeoxycholic acid (UDCA) and TUDCA show neuroprotective properties, which can induce the production of neurons without cytotoxicity and permeability through the BBB. Therefore, they have protective effects on nervous system diseases [81].

#### 5.1.5 SCFAs

The degradation of dietary fiber by intestinal microflora produces a large amount of SCFAs, which can enhance intestinal motility, reduce inflammatory cytokines, and regulate adaptive immune tolerance [58,90]. SCFAs related to brain function include formic acid, propionic acid, butyric acid, valeric acid, and isovaleric acid. These acids participate in nerve transmission, regulate the synthesis of neurotransmitters, and regulate behavior and cognition [46]. Butyric acid and propionic acid can promote tyrosine and tryptophan hydroxylase expressions, which are involved in synthesizing DA, norepinephrine, and 5-HT [19]. Propionic acid can reduce intestinal movement and promote intestinal secretion, while butyric acid can restore the integrity of the blood-brain barrier in aseptic animals (germ-free, *GF*). Butyric acid plays a key role in microglia maturation, dendritic sprouting, an increase in the number of synapses, and the learning process and long-term memory support [46]. Butyrate is the colonic mucosa’s main energy source, maintains human intestinal health, and regulates gene expression, differentiation, inflammation, and apoptosis in host cells [91,92]. Butyrate can also play a neuroprotective role as a histone deacetylase inhibitor [18]. Valeric acid, isovaleric acid, isobutyric acid, and formic acid affect AD pathogenesis by interfering with the microglia and astrocyte activation to help reduce inflammation and the accumulation of Aβ and tau [50]. Therefore, not all microbial metabolites negatively affect the nervous system.

### 5.2 Neurotransmitter

Stress and emotion can cause the brain to release hormones or neurotransmitters that affect intestinal microorganisms’ composition. Intestinal microflora can also affect brain function by producing neurotransmitter precursors or regulating neurotransmitters. Intestinal microflora can synthesize GABA to stimulate 5-HT secretion by chromaffin cells in the intestinal wall and affect the expression of brain-derived neurotrophic factor (BDNF) and DA in the brain [93-95]. GABA, 5-HT, and NMDA are three types of neurotransmitters.

#### 5.2.1 GABA

GABA is a major human inhibitory neurotransmitter produced by *Lactobacillus*, *Bifidobacterium*, and *Bacteroides* [46,96]. It can lead to cognitive and memory impairment when the function of the GABA system is impaired [93]. *Lactobacillus* and *Bifidobacterium* are components of normal intestinal microbiota, which can convert sodium glutamate into GABA [93]. GABA acts on inhibitory synapses in the brain by binding to specific transmembrane receptors on the plasma membrane during presynaptic and postsynaptic transmission. It also participates in the proliferation of precursor neurons, synaptic formation, and inhibition of inflammation *in vivo* [46]. Intestinal bacterial disorders produce intestinal damaging GABA, causing the accumulation of GABA in feces and affecting mood and behavior [68,97]. Animal studies have shown that *Lactobacillus rhamnoides GG* can regulate the expression of GABA receptors in the brain, playing a useful role in treating mood disorders [19]. Therefore, the changes in intestinal flora are closely related to GABA regulation.

#### 5.2.2 5-HT

5-HT is a monoamine neurotransmitter that is produced by intestinal chromaffin cells [8]. It is a key component of the gut-brain axis and plays the role of neurotransmitter in the CNS and intestinal nervous system [93]. More than 95% of 5-HT is synthesized in the intestine and plays an irreplaceable role in the gastrointestinal tract function. *Candida*, *Streptococcus*, *E. coli*, and *Enterococcus* indirectly stimulate intestinal cells to store and release 5-HT [46]. These bacteria play an important role in the synthesis of 5-HT. Linstow et al. used high-pressure liquid chromatography to analyze the 5-HT content in the neocortex, hippocampus, striatum, brainstem, and cerebellum of 18-month-old AD transgenic mice. The levels of all specific regions that could produce monoamine compounds in AD mice changed. The content of 5-HT in the neocortex decreased by 30% and increased by 18% in the brainstem compared with WT mice [98]. Yano et al. found that the content of 5-HT in the blood of GF mice was about 60% lower than that of specific-pathogen-free mice, and the 5-HT concentration significantly increased when reconstructing the intestinal flora of GF mice [99]. *E. coli* and *Enterococci* can produce 5-HT directly and SCFAs indirectly [19].

#### 5.2.3 NMDA

NMDA is an important receptor in the process of learning and memory [100]. Neufeld et al. showed that the NMDA receptor mRNA expression in the hippocampus of aseptic mice decreased significantly. Furthermore, the hippocampal NMDA level decreased significantly after antibiotic treatment, indicating that intestinal flora was involved in the metabolic activity of NMDA [101].

### 5.3 Inflammatory response

The intestinal microbiota is closely related to systemic inflammatory response changes. Under normal circumstances, the immune response in the human body is initiated by microglia and terminated with the removal of pathogens, cell death, other cell fragments, and tissue repair [102]. However, under some pathological conditions, the injury persists, the immune response changes or is damaged, and the chronic inflammatory process may cause damage to neurons. The regulation of immune inflammation is related to the pathogenesis of neurodegenerative diseases [14]. Changes in intestinal microbiota, a biological disorder, may contribute to the neuro-inflammatory process observed by AD [102]. These changes trigger brain inflammation, decrease anti-inflammatory bacteria, and increase pro-inflammatory bacteria in the AD brain [103]. Intestinal microbiota can also enhance the inflammatory response to Aβ in the brain by activating the host’s innate immune system, leading to neuroinflammation [48]. Jeffrey Cummings, a professor at Cleveland Medical Center and winner of the Bengt Winblad Lifetime Achievement Award (2018) of the American Alzheimer’s Association, stated that “The latest study found that the microbiome in AD is abnormal, and the abnormal microbiome will stimulate the release of peripheral inflammatory cells into the brain, thereby promoting neuro-inflammation.” Therefore, inflammation is beneficial or harmful to the brain, depending on the activation intensity at different stages of neurodegeneration [14]. The following sections introduce substances that can cause inflammation and explain how they affect AD development by regulating intestinal flora.

#### 5.3.1 Pro-inflammatory cytokines

Pro-inflammatory cytokines include interferon, transforming growth factor-β (TGF-β), tumor necrosis factor-α (TNF-α), nitric oxide, IL-1, IL-6, and IL-17 [104,105]. Pro-inflammatory cytokines participate in all aspects of effector cell activation in the inflammation process by participating fully in the differentiation, maturation, chemotaxis, retention, and sensitization of effector cells [104]. Moreover, they impact systemic immunity and inflammatory response [106]. Several studies have shown that the pro-inflammatory cytokine levels in AD patients, such as IL-1β and TNF-α, are abnormally elevated [14]. Activated microglia are also involved in the secretion of pro-inflammatory cytokines, such as IL-1, IL-6, TNF-α, and TGF-β, promoting neurological disease progression [14,107]. In a study of the relationship between intestinal bacteria, systemic inflammation, and AD, Cattaneo et al. highlighted the relationship between cognitive impairment, brain amyloidosis, and inflammatory markers circulation [47]. It was found that the abundance of *E. coli*/*Shigella* and *Bacteroides fragilis* in the plasma of patients with amyloidosis was increased significantly, and the number of rectal bacteria was significantly decreased. Furthermore, the pro-inflammatory cytokine (IL-6, CXCL2, NLRP3, and IL-1β) levels in the plasma were significantly increased, and the anti-inflammatory cytokine (IL-10) level was decreased. Therefore, the changes in intestinal microbiota (the increase of pro-inflammatory bacteria [*E. coli*/*Shigella*] and the decrease of anti-inflammatory bacteria [*rectal fungi*]) may be related to the cognitive impairment of AD [47,61,63]. In addition, microbial disorders at the intestinal level may damage intestinal permeability and induce systemic activation of the immune system [61]. Microbial disorders and increased intestinal permeability may increase the IL-6 level in the body and trigger an inflammatory response [103]. These results suggest that the intestinal flora changes are closely related to the host’s inflammatory state and cognitive impairment.

#### 5.3.2 Bacterial amyloid protein

**Figure 3.**
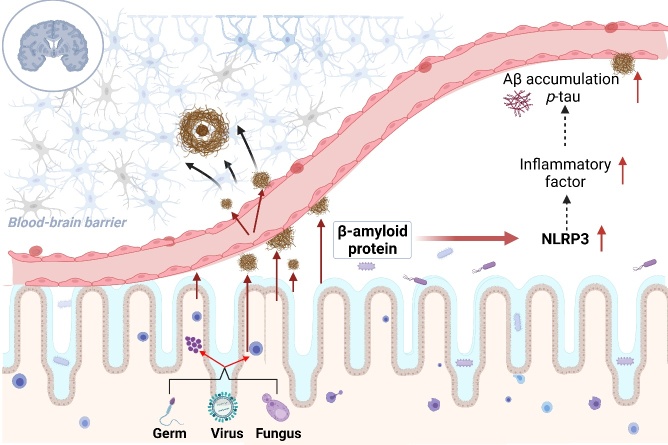
**Mechanism of bacteria-derived amyloid protein in inflammation regulation in Alzheimer’s disease.** NLRP3: NOD-like receptor protein 3.

With the increase of age, the balance between the microbial ecosystem and the immune system in the human body is disrupted, and microorganisms stimulate intestinal mucosal epithelial cells and lymphoid tissue to release amyloid protein [46]. Most microorganisms in the human body, including bacteria and fungi, secrete functional amyloid [108]. The amyloid produced by microorganisms may contribute to amyloidosis in the CNS of AD patients [108]. Microorganism-derived amyloid may cause Aβ aggregates to cross-sow, initiating prion-like reproduction [109]. It also induces inflammation and leads to AD [110]. In the regulation of inflammation, amyloid protein of microbial origin plays the role of PAMP [108,111], which can cross the damaged blood-brain barrier and enter the blood flow to the CNS, where it is deposited in the brain. This mechanism promotes the aggregation and nucleation of β-amyloid protein and accelerates AD progression [27]. Microorganisms, including bacteria, fungi, and viruses, stimulate intestinal mucosal epithelial cells and lymphoid tissue to release amyloid protein [46,108]. Bacterial amyloid protein can cross the blood-brain barrier into the blood flow to the central nervous system, deposit in the brain, and promote β-amyloid protein accumulation [27]. Bacterial amyloid protein can also cause the NLRP3 inflammatory bodies activation, promote the microglia activation, release inflammatory factors, and promote the aggregation of Aβ and *p*-tau [112-114] (Fig. 3). The known bacteria that can produce functional extracellular amyloid fibers are *E. coli, Salmonella enterica, S. typhimurium, Bacillus subtilis, Mycobacterium tuberculosis*, and *Staphylococcus aureus* [27]. Among them, *E. coli* endotoxin forms Aβ fibers in vitro, suggesting that it is involved in AD pathogenesis [14,48].

### 5.4 Immune cells

**Figure 4.**
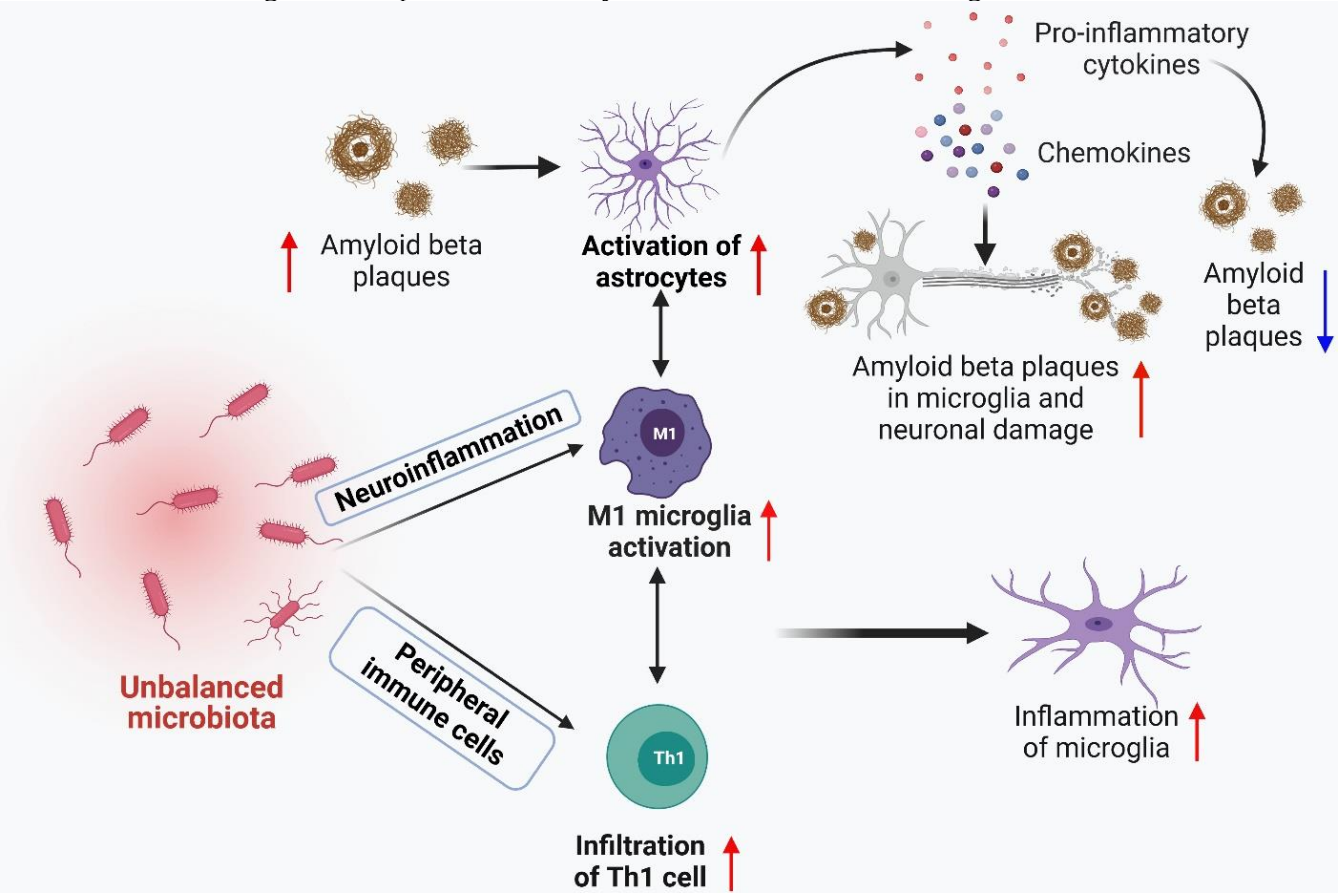
**Effect of gut microbiota on immune cells in Alzheimer’s disease.** Intestinal dysbacteriosis can activate microglia and promote Th1 cell infiltration. It makes these two interact with M1 microglia locally, triggering the differentiation of microglia to a pro-inflammatory state, resulting in cognitive impairment.

Intestinal microflora can change immune cells’ frequency, function, and transport mode by changing the inflammatory degree of macrophages, lymphocyte surface proteins, and microglia. Therefore, the dynamic balance of peripheral and central immunity is formed [20]. Intestinal microflora affects microglia activation (Fig. 4). Michael Heneka, professor of neuro-degenerative diseases and geriatric psychiatry at the University Hospital in Bonn, Germany, introduced the relevant situation of AD immune system research at the AAIC conference. He stated that the microbiota regulated and affected the immune system. Microglia were activated by this factor, interacted with astrocytes and neurons, and induced neuro-inflammation.

According to Wang et al., microglia activation can be divided into pro-inflammatory M1 and neuroprotective M2. Flow cytometry detected the activated M1 and M2 microglia in the brain homogenate of 5xFAD mice. It was found that M1 and M2 microglia increased in 2-3-month-old 5xFAD mice. M1 microglia continued to increase in the following months, while M2 microglia decreased and maintained a low level at 3-5 months of age [38]. Therefore, the inflammatory response is caused by M1 microglia. In addition, activated astrocytes are supportive cells that provide nutrition and metabolic maintenance for neurons and affect neuroinflammation in AD [115].

Several transgenic mouse models have shown that activated astrocytes accumulate in the brain before any plaque or entanglement pathology is observed, suggesting that astrocyte activation may be involved in AD pathogenesis [115]. Astrocytes outnumber microglia in the brain and have a greater effect on long-term neuro-inflammation. They also secrete pro-inflammatory cytokines and chemokines to clear the accumulated Aβ. The additional deposition of Aβ leads to a positive feedback loop, promoting the astrocytes’ activation and releasing more pro-inflammatory cytokines [115]. The release of a large number of pro-inflammatory cytokines can damage microglia, reduce the ability of microglia to clear toxic Aβ, decrease the synaptic remodeling ability of microglia, and lead to irreversible neuronal damage [115]. Abnormal levels of pro-inflammatory cytokines in plasma reach the brain through the blood-brain barrier and trigger inflammation and astrocyte activation by changing the maturation of microglia [116].

Intestinal microbiota damage can aggravate intestinal inflammation and reduce the expression of tight junction proteins in colonic epithelial cells. A decrease in the junction can cause microbial exudates to enter the circulatory system, triggering an inflammatory response. Therefore, intestinal microflora is important for microglia maturation and inhibition of inflammation in the CNS [103]. Microglia-induced neuro-inflammation can be used as a target for developing anti-AD drugs [53]. Moreover, the key inflammatory components in the pathogenesis of AD should be further explored to develop better methods for treating this disease [14].

## 6. Antibiotics

Antibiotic therapy may combat AD by changing the intestinal microflora [117]. DNA analysis of the cecum and feces of mice treated with antibiotics showed that Aβ plaque deposition was significantly reduced and could restore intestinal microflora similar to that of the control group. Furthermore, intestinal permeability was also restored, and glial cell reactivity in the local area of the plaque was weakened [21,61,117]. Ceftriaxone use can reduce the increase of glutamate by improving glutamate transport, which is usually present in the area of Aβ plaque deposition, thereby improving neuronal activity in APP/PS1 transgenic mice [118]. Wang et al. also found that the abundance of intestinal microflora in 5xFAD mice significantly decreased after treatment with ABX, a mixture of antibiotics containing ampicillin, streptomycin, and colistin. Flow cytometry was used to detect the frequency of Th1 cells and M1 microglia in the brain homogenate of mice. The Th1 cells and M1 microglia were decreased in the brain. These findings proved that the use of antibiotics could relieve the symptoms of AD [38].

However, some antibiotics (such as streptozotocin and ampicillin) can disrupt the intestinal bacteria balance [119]. The use of these antibiotics is conducive to or worsens the disease process. For example, rats taking ampicillin have elevated glucocorticoids, increased anxiety-like behavior, and impaired spatial memory [102]. The increase in glucocorticoids is related to memory impairment and decreased hippocampal BDNF, common features of AD pathology. Ampicillin treatment also significantly decreases the level of NMDA receptors in the hippocampus of rats [102]. In addition, long-term treatment of APPswe/ps1ΔE9 mice with broad-spectrum combination antibiotics can lead to long-term intestinal microbial composition and diversity disorders. It includes different concentrations of gentamicin, vancomycin, metronidazole, neomycin, ampicillin, kanamycin, colistin, and cefoperazone. It was accompanied by a peripheral inflammatory environment and pathological changes of Aβ plaque [45,120] (Table 4).

**Table 4:** Effects of antibiotics on the mouse model of AD.

Antibiotic	Target	Effects	References
**Ceftriaxone**	Glutamic acid	-Reduced glutamate transport -Improve neuronal injury	[118]
**-Ampicillin** **-Streptomycin** **-Colistin**	-Th1 cell -M1 microglia cell	-Decreased abundance of intestinal flora -Decrease of Th1 cells and M1 microglia cell	[38]
**Streptozotocin**	Gram-positive bacteria	-Memory deficits	[102, 119]
**Ampicillin**	- Gram-positive bacteria - Gram-negative bacteria	-Increased glucocorticoid -Increased anxiety behavior -Impaired spatial memory -Decreased NMDA receptor in hippocampus -Memory impairment	[102]

Antibiotics with selective antibacterial activity should be developed. A key factor is the identification of intestinal microbiota associated with the disease. Therefore, the future of antibiotics as drugs for treating AD depends on the research progress on the role of intestinal microbiota [102]. The choice of antibiotics to treat AD and other neurodegenerative diseases should be carefully evaluated in humans due to controversial results in some clinical trials. Presently, there is a lack of scientific basis for using antibiotics for treating AD [102]. Probiotics and antibiotics can be used to counteract the negative effects of antibiotics.

## 7. Probiotic bacteria

Probiotics are living microorganisms, and their use is a new and safe way to maintain healthy intestinal flora. They can inhibit other microorganisms’ growth or improve intestinal flora’s function by competing with other intestinal microorganisms for receptors and binding sites on the intestinal mucosa [14,121]. Recently, probiotics have been used to treat many gastrointestinal and neurodegenerative diseases, including AD [18,39].

Akbari et al. evaluated the effects of probiotic supplements (*Lactobacillus acidophilus, L. casei, Bifidobacterium*, and *L. fermentum*) on cognitive function and metabolic status in 60 patients with AD [122]. It was found that the score of the mini-mental state examination was significantly higher in the probiotic intervention group than in the control group [18,39,120]. In animal models, Liu et al. found that probiotics can affect the CNS through the gut-brain axis [123]. *L. plantarum* restores acetylcholine levels and improves cognitive impairment in AD rats [39]. Distrutti et al. changed the intestinal flora composition and improved the function of neurons in aged rats by using VSL3 [124]. VSL3 is a probiotic mixture composed of eight Gram-positive bacteria, including *Streptococcus thermophilus, Bifidobacterium longum, L. acidophilus*, and *L. plantarum* [124]. Aged rats treated with VSL3 showed slightly decreased microglia activation markers, reduced pro-inflammatory factor production, and increased M2 macrophage activity. Furthermore, the rats also showed increased expression of inflammation and neuronal remodeling genes, such as BDNF and synaptophysin [18]. VSL3 can also regulate the expression of inflammatory cytokines, reduce oxidative stress, and improve nutritional status [14]. Probiotics may improve intestinal microflora composition, but they do not show a permanent effect in treating flora disorders [18].

*Lactobacillus, Lactococcus*, and *Bifidobacterium* are the most-studied probiotics [55]. With the revelation of the microbial-brain relationship, recent studies have delved into the link between probiotics and neuronal health, especially in AD [55]. Therefore, altering intestinal microflora through specific probiotic strains may naturally alleviate the pathophysiology or symptoms of neurodegenerative diseases such as AD. Researchers have recently demonstrated a new oral preparation of *Lactobacillus* and *Bifidobacteri*a (SLAB51). It will combat AD progress in 3xTg-AD mice and changes in intestinal flora [125]. The results showed that administrating SLAB51 significantly increased the number of *Bifidobacteria* and decreased the number of campylobacters (*Helicobacter pylori*) in AD mice compared with WT mice [126]. Examination of the metabolites of these bacteria showed acetic acid, propionic acid, and butyric acid increased significantly in AD mice after SLAB51 treatment [126]. Subsequently, the level of pro-inflammatory cytokines decreased in AD mice after administrating SLAB51. It confirmed that the changed microflora had anti-inflammatory effects [126]. In addition, adenosine monophosphate-activated protein kinase (AMPK) and protein kinase B (Akt) mediated increased glucose metabolism in the brain. It is another mechanism by which SLAB51 inhibits the progression of AD [125].

**Figure 5.**
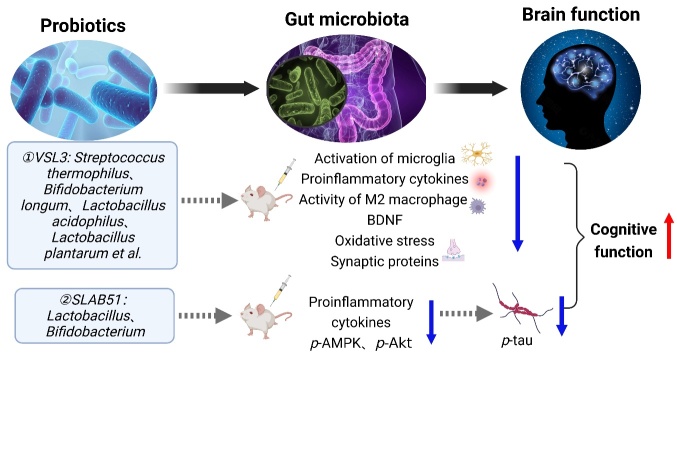
**Different prebiotic therapies and their effects on gut microbiota and brain function.** Aged rats treated with VSL3 decreased the microglial activation and pro-inflammatory factors production and increased the activity of M2 macrophages. It also showed increased expression of genes related to inflammation and neuronal remodelings, such as BDNF and synaptophysin, decreased oxidative stress, and improved cognitive function. SLAB51 can reduce the pro-inflammatory cytokine, *p*-AMPK, and *p*-Akt levels in AD mice, decrease the *p*-tau level in the brain of mice, and improve their cognitive function. BDNF = brain-derived neurotrophic factor.

Previous studies have found that impaired glucose uptake and metabolism in the brain of AD patients are due to decreased levels of glucose transporter 1 (GLUT1) and GLUT3 [127]. It results in impaired brain glucose uptake and tau protein hyper-phosphorylation, a major pathological marker of AD [125]. The dysfunction of AMPK and Akt are the key receptors of glucose metabolism and can lead to energy metabolism disorders and glutamate gene expression regulation and is related to tau protein hyperphosphorylation [128]. Tau is one of the main characteristics of AD [125]. Glutamate levels decrease and phosphorylated AMPK and Akt increase in the brains of older AD animals. A previous study demonstrated that probiotics could reduce *p*-tau by restoring glutamate expression levels in the brain and reducing the phosphorylation of key metabolic regulators such as AMPK and Akt [125]. In AD animals treated with SLAB51, the p-AMPK and p-Akt levels increased, the *p*-tau level in the mouse brain decreased significantly, and the number and size of hyperphosphorylated tau aggregates were reduced [125]. The results showed that SLAB51 probiotics changed the intestinal flora of 3xTg-AD mice, reduced the aggregation and phosphorylation of tau protein, alleviated brain injury, improved cognitive ability, and delayed the progress of AD [39,125]. The effects of VSL3 and SLAB51 on intestinal flora and brain function are summarized in Figure 5.

However, not all probiotic interventions have a positive effect. Agahi et al. conducted a double-blind clinical trial to evaluate the effect of probiotic treatment on AD [129]. After 12 weeks of treatment with a placebo and probiotics in the control group and severe AD patients, no significant difference was observed in cognitive tests, serum inflammation, and oxidation biomarkers between AD and control group patients [120]. This finding suggested that the cognitive and biochemical status of patients with severe AD is not sensitive to probiotic supplements. Other studies reported that modifying human intestinal bacteria with probiotic supplements did not change cognitive function or inflammation in severe AD patients [39]. The results of Akbari et al. [130] showed that the efficacy of probiotics in treating AD depends on the severity of the disease and requires larger-scale research [120,129].

Recent studies have hypothesized that supplementing multiple probiotics may beneficially improve AD pathology [131,132]. Leblhuber et al. showed that supplementation with multiple probiotics could change the intestinal bacteria composition and the metabolism of Trp in the serum of AD patients, indicating that it activates the immune system [61,131]. Francesca et al. studied healthy dietary patterns characterized by a high intake of various probiotics. The results showed that probiotic dietary supplements could delay neurocognitive decline and reduce the risk of AD [133]. Probiotic supplementation affects normal brain activity [134] and significantly improves the cognitive ability of AD patients [102,122]. In addition to using probiotics to treat AD, Abraham et al. proposed regular physical exercise as adjuvant therapy to study the effects of exercise and probiotic intervention on the occurrence of AD in APP/PS1 transgenic mice [135]. It was found that exercise, probiotics, and their combination reduced the number and area of amyloid plaques in the hippocampus of mice, partly due to changes in intestinal microbiota [61,135].

Probiotics help human bodies obtain the substances necessary to prevent inflammation and related diseases [102]. The whole response chain occurs only when the intestinal flora is in equilibrium [102]. Sufficient amounts of probiotics should be consumed throughout the diet to maintain this balance. The most common probiotics are *Bifidobacterium* and *Lactobacillus* strains, found in foods such as yogurt, fermented cheese, vegetables, or dietary supplements [102]. Various microflora can be obtained through diet [102]. However, poor eating habits, the use of antibiotics, and stress may impair the bacteria activity or change their composition, leading to an imbalance of intestinal flora that puts health at risk [102]. Therefore, the effects of probiotics in preventing or controlling AD need to be further studied [136].

## 8. Discussion

Evidence shows that intestinal microbiota is closely related to the occurrence of AD. The definition of a healthy microbiome may be a major problem in microbiome medicine. The human body contains a large number of various bacteria, which vary in each individual, making the research of the microbiome challenging. Therefore, personalized medical methods should be developed in the future. At the laboratory level, more research is needed to fully understand the internal and external factors that limit microflora changes by diet or other interventions. To better describe the composition of microorganisms, the genera and the strain level should be studied. Metabonomics, proteomics, and even genomics methods can be researched further in addition to 16S rRNA gene sequencing. Systems biology methods will be the key to integrating these multiple data sets.

Chen et al. used a groundbreaking key association pair study of changes in the intestinal flora and brain metabolism during an animal’s lifetime using a dual recombination platform. The study screened four pairs of association pairs: “lipid-*Spirochete*,” “free fatty acid-*Firmicutes*,” “bile acid-*Firmicutes*,” and “neurotransmitter-*Bacteroides*” [137]. However, animal associations need to be carefully verified in the human body. More information can be obtained from the blood and fecal metabolic groups and fecal microbiome to confirm the link between the brain metabolic group and the intestinal microbiome. It would establish a system and network connecting the brain and the intestinal tract.

In addition to the bacteria and metabolites relationship, other associations should be researched. Chen et al. provided a new idea for association research and further deepened the understanding of the “gut-brain axis.” Symbiotic bacteria are another research target. Wang et al. raised WT and 5×FAD mice in a cage. They shared intestinal flora through feces, resulting in similar changes in the composition of intestinal microorganisms and cytokines in the brain [38]. In addition, research beyond bacteria must be expanded, especially in the field of virosomes and bacteriophages, to fully understand the importance of microflora in regulating brain function. Moreover, the molecular mechanisms involved in the two-way microbial-intestinal-brain communication should be explained to identify and understand the role of produced metabolites and their potential interactions with the host.

Presently, pre-clinical studies are based on animal models of AD, supported by a small number of clinical trials, because it is difficult to create hypotheses and ideas in this area. Nevertheless, the drugs currently available on the market and other drug candidates that are still in clinical trials are designed and studied by taking the brain as the main target. Due to this, antibiotics and probiotics stand out in many studies and are expected to combat AD effectively.

The use of antibiotics for therapeutic or prophylactic interventions in patients with AD is intriguing because it is relatively cost-effective and can be combined with specific dietary regimens and probiotics. Presently, the research in this field is making great progress, but the clinical application is still limited. It is because the role of antibiotics in AD may be extensive but depends on the antibiotic type. Therefore, antibiotics with selective antibacterial activity should be developed, and the most important intestinal microbiota associated with AD needs to be identified. Current studies have shown some intestinal flora changes in AD animals or patients, but these are inconsistent.

Probiotics meet in the case of antibiotics. The advantage is that it does not contain chemicals, and the disadvantage is that the location is unclear. It should be prepared following pharmacopeia standards if it is used as medicine. However, the content of bacteria in the pharmacopeia is not clear. If probiotics are used as health products or medical food, manufacturing companies may have some difficulties preparing these products, and their practical interests should be considered. Thus, this is still an issue worthy of discussion. In addition, compared with classic drugs, probiotics have a lower risk of side effects, which is their biggest advantage and should increase the interest of pharmaceutical companies to participate in developing these products.

Besides drugs, antibiotics, and probiotics, diet may be a major factor affecting microflora’s composition. Because the diet is rich in many nutrients, the metabolic pathway in the body also covers the whole body. People may be more willing to eat to improve the disease than to take antibiotics or probiotics. Thus, all human studies should have good dietary data to better understand the relationship between diet, microbiome composition, and the brain. The effects of dietary composition and metabolites produced by microorganisms on host physiology and health are attracting increasing attention, which is particularly important in promoting the development of non-drug therapy.

In addition, infection is also closely related to AD. Some researchers have suggested that herpes simplex virus type 1 (HSV1) is a strong risk factor for AD. It was reported that Aβ plaques accumulated in HSV1-infected cell cultures and mouse brains [138], and the risk of dementia significantly increased in patients infected with HSV, which can be reduced after antiherpes drug treatment [139]. In addition, studies have shown that chronic spirochete infection can lead to brain atrophy and amyloid deposition, which cause progressive dementia, and various types of spirochetes can be detected in the brain tissues of AD patients, including *B.Burgdorferi* and six kinds of periodontal pathogenic spirochetes (*T.scranskii, T.ectinovorum, T.denticola, T.media, T.mylovorum, and T.amerlovorum.*) [140]. It was determined that spirochete induction *in vitro* could increase the level of Aβ, Aβ deposition, and tau phosphorylation, which significantly correlated with AD [140]. AD is also significantly associated with *cytomegalovirus*, *herpesviridae*, *Chlamydia pneumoniae*, and *Helicobacter pylori* [141]. These pathogens can not only escape the destruction of the host immune system, leading to persistent infection and increasing the expression of pro-inflammatory factors, but activate the immune system, leading to Aβ deposition, tau protein phosphorylation, neuronal damage, and apoptosis [141]. To summarize, both microbial and viral infections are inextricably linked with AD.

In addition, changes in drinking [142], smoking [143], exercise [46], circadian rhythm [144], and sleep [145] also affect the composition of microflora. AD is a complex chronic disease whose etiology is complicated, making research challenging. Therefore, many possible treatment strategies have been put forward in the research process. This study proposed an innovative strategy in AD treatment, which involves a long-term dietary adjustment in conjunction with drug treatment and adjuvant therapy with probiotics or antibiotics to regulate intestinal flora. The implementation of this idea needs to be done in the future, and several aspects need to be studied.

First, research needs to be done to determine when the intestinal flora will change and whether it is before or after the onset of AD. Second, the role of metabolites of intestinal microbiota, such as SCFAs, in AD needs to be studied. Third, an investigation needs to be conducted to examine whether neurotransmitters of the intestinal microbiota are involved in the central pathological process of AD. Finally, a study should be done on how external interventions can accurately reconstruct the balance of micro-ecosystems to treat AD. In clinical trials, more emphasis must be placed on antibiotics, probiotics, and potential fecal microbiome transplantation interventions. Although the composition and distribution of different animal species are similar to those of human intestines, there are significant differences in the complexities of animal and human intestinal microbiota. Therefore, more clinical studies are needed to understand the mechanisms.

Intestinal microbiota, known as a “forgotten organ,” is a new area for research on AD and other neurodegenerative diseases. With deeper research, the pathological mechanisms of intestinal biota will be gradually revealed. Furthermore, progress may be made in the early diagnosis and development of new therapeutic targets and drugs for AD treatment.
